# Facile Spray-Coating of Antimicrobial Silica Nanoparticles for High-Touch Surface Protection

**DOI:** 10.1021/acsami.4c18916

**Published:** 2025-02-12

**Authors:** Carolina Duarte Bernardino, Mihyun Lee, Qun Ren, Bastian Ruehle

**Affiliations:** † Federal Institute for Materials Research and Testing (BAM), Richard-Willstaetter-Strasse 11, D-12489 Berlin, Germany; ‡ Humboldt University Berlin, Unter den Linden 6, D-10117 Berlin, Germany; § Laboratory for Biointerfaces, Empa, Swiss Federal Laboratories for Materials and Technology, Lerchenfeldstrasse 5, 9014 St. Gallen, Switzerland

**Keywords:** mesoporous silica nanoparticles, thin films, antimicrobial coatings, spray-coating, infectious diseases, pathogen transmission, high-touch surfaces

## Abstract

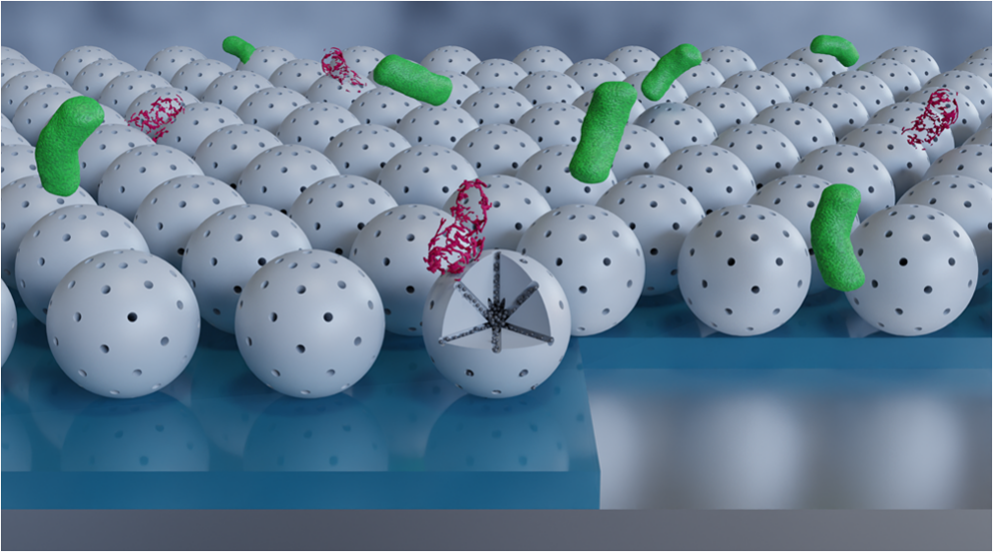

The rising threat from infectious pathogens poses an ever-growing challenge. Metal-based nanomaterials have gained a great deal of attention as active components in antimicrobial coatings. Here, we report on the development of readily deployable, sprayable antimicrobial surface coatings for high-touch stainless steel surfaces that are ubiquitous in many healthcare facilities to combat the spread of pathogens. We synthesized mesoporous silica nanoparticles (MSNs) with different surface functional groups, namely, amine (MSN-NH_2_), carboxy (MSN-COOH), and thiol groups (MSN-SH). These were chosen specifically due to their high affinity to copper and silver ions, which were used as antimicrobial payloads and could be incorporated into the mesoporous structure through favorable host–guest interactions, allowing us to find the most favorable combinations to achieve antimicrobial efficacy against various microbes on dry or semidry high-touch surfaces. The antimicrobial MSNs were firmly immobilized on stainless steel through a simple two-step spray-coating process. First, the stainless steel surfaces are primed with sprayable polyelectrolyte solutions acting as adhesion layers, and then, the loaded nanoparticle dispersions are spray-coated on top. The employed polyelectrolytes were selected and functionalized specifically to adhere well to stainless steel substrates while at the same time being complementary to the MSN surface groups to enhance the adhesion, wettability, homogeneity, and stability of the coatings. The antimicrobial properties of the nanoparticle suspension and the coatings were tested against three commonly found pathogenic bacteria, *Staphylococcus aureus*, *Pseudomonas aeruginosa*, and *Escherichia coli*, as well as a fungal pathogen, *Candida albicans*. Especially MSN-SH loaded with silver ions showed excellent antimicrobial efficacy against all tested pathogens under application-relevant, (semi)dry conditions. The findings obtained here facilitate our understanding of the correlation between the surface properties, payloads, and antimicrobial activity and show a new pathway toward simple and easily deployable solutions to combat the spread of pathogens with the help of sprayable antimicrobial surface coatings.

## 1 Introduction

High-touch surfaces are known to play an important role in the transmission of infectious diseases.^1^ It has even been shown that a single touch of human skin on a contaminated surface can lead to the spread of a pathogen.^2^ In a hospital environment, high-touch surfaces near patients pose the greatest risk of transmitting healthcare-associated infections.^3^ The most easily transmitted pathogens from inanimate surfaces to the skin are *Escherichia coli*, *Salmonella spp.*, *Staphylococcus aureus*, *Candida albicans*, *Pseudomonas aeruginosa*, rhinoviruses, HAV and rotaviruses.^2^

Today, the cleaning and disinfection of contaminated surfaces are often done using conventional methods whose success relies on how thoroughly a disinfectant or cleaning agent is applied to the contaminated surface, on the disinfectant itself, on the type of contamination, and on the contact time. Some studies also reveal that disinfected surfaces continue being contaminated by pathogens.^4^ Most commercially available antimicrobial cleaning and/or disinfection agents focus on the direct and immediate inactivation or destruction of micro-organisms; however, it is well known that the disinfection of surfaces does not last long, and even multiple rounds of disinfection have been shown to be insufficient to eliminate some pathogens.^5^ In addition, Kramer et al. found that many bacteria (Gram-positive and Gram-negative), mycobacteria, and spores can persist for months on dry surfaces.^6^ Stephens et al. reported the exchange of microbes through surfaces^7^ and noted in their study that depending on environmental factors and the type of material, both viruses and pathogenic bacteria can survive on the surfaces for days. Besides these challenges for disinfecting contaminated surfaces with conventional disinfectants, some reports have also suggested that the overuse of alcohol-based hand sanitizers in hospital settings is a driver of resistant strains of pathogens.^8,9^ Microbial resistance is also caused by excessive use of surfactants, hydrogen peroxide, and alkyl chlorides.^9^ This highlights the need for a more reliable and longer-lasting solution to the problem of the transmission of pathogens through surfaces.

In response to these issues, antimicrobial surfaces and coatings designed to minimize the presence of pathogens are being studied for use in various settings, including healthcare centers, long-term care facilities, public transport, schools, and businesses, to reduce human exposure and help prevent the spread of infectious pathogens.^10^ Extensive research has focused on finding solutions to prevent bacterial transmission and biofilm formation by killing or reducing the number of microbes. These solutions include antimicrobial metallic surfaces, surface-bound active antimicrobials, biocidal coatings, and passive pathogen-repellent surfaces through chemical modifications and micro- and nanostructuring of the surface.^11^

Metallic surfaces, such as silver and copper, show high antimicrobial efficiency against a broad spectrum of microbes.^12^ However, bulk metal coatings can be expensive because they require large quantities of pure metal. Additionally, the formation of an oxidative layer on metallic surfaces can significantly reduce antimicrobial efficacy over time, limiting their effectiveness to the short term.^13,14^ Furthermore, these coatings are typically produced by electroplating, thermal spray, or chemical/physical vapor deposition, which cannot be easily renewed on a regular basis on surfaces that are currently in use.^15^ On the other hand, spray-coating of antimicrobial substance in an innocuous medium, such as water or ethanol, allows for easy renewal of the coatings by untrained individuals due to the simplicity of the process.^16^ Despite the convenience and potential for long-term maintenance of antimicrobial effects, currently, there are only a limited number of sprayable products available for antimicrobial coatings, primarily based on quaternary ammonium compounds.^17^

Nanomaterials are an interesting alternative to bulk metal surfaces and have already been explored as carriers for antimicrobial compounds such as metal ions/nanoparticles (silver, copper, or zinc), antibiotics, and antimicrobial proteins/peptides. They have demonstrated improvements in several aspects, including reduced cytotoxicity, sustained antimicrobial activity, targeted delivery, and controlled release of antimicrobial substances compared to the compounds without the carriers.^13,18^ Most of these materials have however been developed for biomedical applications such as antibiotic therapeutics, with very different requirements compared to sprayable surface coatings required to work on (semi)dry high-touch surfaces.

In the context of triggered and sustained release of antimicrobially active payloads, mesoporous silica nanoparticles (MSNs) are very strong candidates, due to their biocompatibility, well-defined morphology, mesoporous structure, high porosity, chemical inertness, and relative ease of synthesis.^19^ This type of mesoporous nanoparticles possesses both external (outside) and internal (pore wall) surfaces that can be functionalized with several organic or inorganic groups.^20^ Moreover, MSNs are popular and efficient drug carriers due to their large loading capacity and the possibility for spatiotemporally controlled, triggered release.^21^ Due to their high surface area per unit mass and large number of functional groups, MSNs also show high adsorption capacity for metal ions.^22^ Based on these properties, MSNs loaded with antimicrobially active silver or copper ions are promising candidates for antimicrobial surface coatings.

The antimicrobial properties of silver and copper are in part based on reactive oxygen species (ROS) generation. ROS can damage cellular components such as lipids, proteins, and DNA, leading to microbial cell death.^11,23^ Additionally, the interaction of Ag^+^ with phospholipid tails and proteins containing cysteine or methionine plays an essential role in bacterial cell wall disruption.^11^ However, many silver- and copper-based products face challenges with uncontrolled release of metal ions, toxicity, and stability or wear resistance, whereas MSNs loaded with antimicrobially active metal ions can provide controlled and sustained release of these ions. Furthermore, MSNs can be specifically functionalized with surface functional groups tailored toward favorable interactions with these ions, potentially allowing more selective antimicrobial activity.

**Figure 1:**
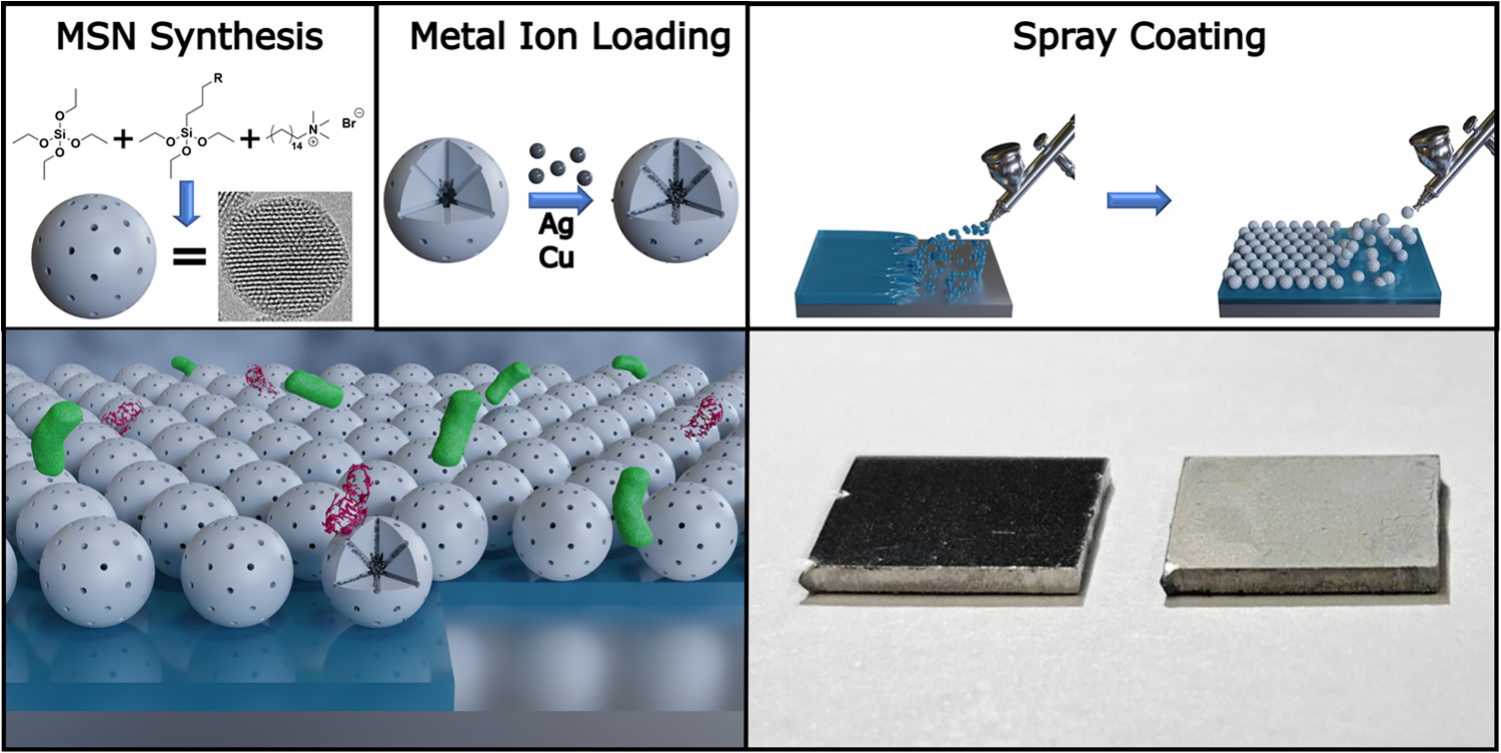
Schematic representation of the steps described in this work for creating the antimicrobial surface coatings (gray: MSN, black: metal ions; silver: stainless steel substrates; blue: adhesion layer; green: live microbes; red: dead microbes). Top: mesoporous silica nanoparticle synthesis, metal ion loading, and deposition on an adhesion layer on stainless steel substrates via spray-coating. Bottom: schematic representation (side view) of the metal-ion-loaded nanoparticles on top of an adhesion layer on a stainless steel substrate interacting with microbes, and a real photograph of a non-spray-coated (left) and a spray-coated stainless steel substrate (right).

In this study, we investigate the influence of different surface functionalization and metal ion loading of MSNs on the antimicrobial efficacy of readily deployable, sprayable antimicrobial surface coatings (Figure 1). MSNs with different surface functional groups are synthesized and loaded with antimicrobial active silver and copper ions to endow them with antimicrobial activity when used in sprayable water- or ethanol-based formulations. The antimicrobial efficacy of these materials has been successfully demonstrated and optimized under wet conditions relevant to those applications. However, when applying such MSNs to high-touch surfaces, the environment where pathogens are exposed to the MSNs can be very different, as these surfaces are usually kept dry or semidry. This results in a lower release of metal ions compared to wet conditions (in a solution). In this context, optimizing the interaction between metal ions and MSNs is crucial to achieve sufficient antimicrobial effects under the dry or semidry condition considering that the interaction of metal ions with bacterial phospholipid tails and proteins containing cysteine or methionine is essential for microbial killing through cell wall disruption.^10^ The functional groups on the internal and external surfaces of the MSNs are expected to have a large influence on the loading and release capacity of the different metal ions investigated in this study. Strong interactions between metal ions and MSNs can lead to high ion loading but may limit the mobility and release of metal ions, potentially resulting in insufficient antimicrobial activity of the coating. Conversely, weak interactions could result in a low loading and a burst release within a short period. This means that there will likely be a trade-off between metal ion loading, metal ion leaching, and antimicrobial activity of the coatings, depending on the strength of the interaction of the surface functional groups with the metal ions.

To find this balance, we first investigated how different surface chemistries of MSNs impact the loading and release of metal ions through a simple absorption process and how this correlates with antimicrobial efficacy. Silver and copper ions were chosen as the antimicrobial agents due to their well-documented antimicrobial efficacy.^10,17,18^ MSNs with three different surface functional groups, namely, carboxy groups (MSN-COOH), amine groups (MSN-NH_2_), and thiol groups (MSN-SH), were prepared and compared in terms of metal ion release and antimicrobial efficacy before and after immobilization on a surface. We then demonstrated a simple spray-coating method for firmly attaching the metal ion-loaded MSNs to stainless steel, a model high-touch surface. Since the wetting of stainless steel and nanoparticle adhesion were rather poor when spray-coating the substrates with nanoparticle dispersions directly, we investigated the use of sprayable polyelectrolyte adhesion layers specifically tailored to each type of nanoparticle. These were carefully selected to adhere well to stainless steel substrates and at the same time interact strongly with the MSN surface groups. That way, the adhesion, wettability, and coating homogeneity of our sprayable formulations could be greatly improved. Finally, we tested the effectiveness of our coatings against four model pathogens that play crucial roles in nosocomial infections: *E. coli*, *P. aeruginosa*, *S. aureus*, and *C. albicans* under application-relevant, semidry conditions in simulated touch tests.

## 2 Results and Discussion

We show the development and physical-chemical characterization of functionalized MSN that can be loaded with antimicrobially active metal ions as parts of sprayable formulations, investigate the use of sprayable polyelectrolyte solutions tailored toward the nanoparticle surface functionalization to enhance coating homogeneity and nanoparticle adhesion, and demonstrate the effectiveness of the spray-coated surface coatings against four model pathogens: *E. coli*, *P. aeruginosa*, *S. aureus*, and *C. albicans*.

### 2.1 Functionalized Nanoparticle Synthesis and Characterization

**Figure 2:**
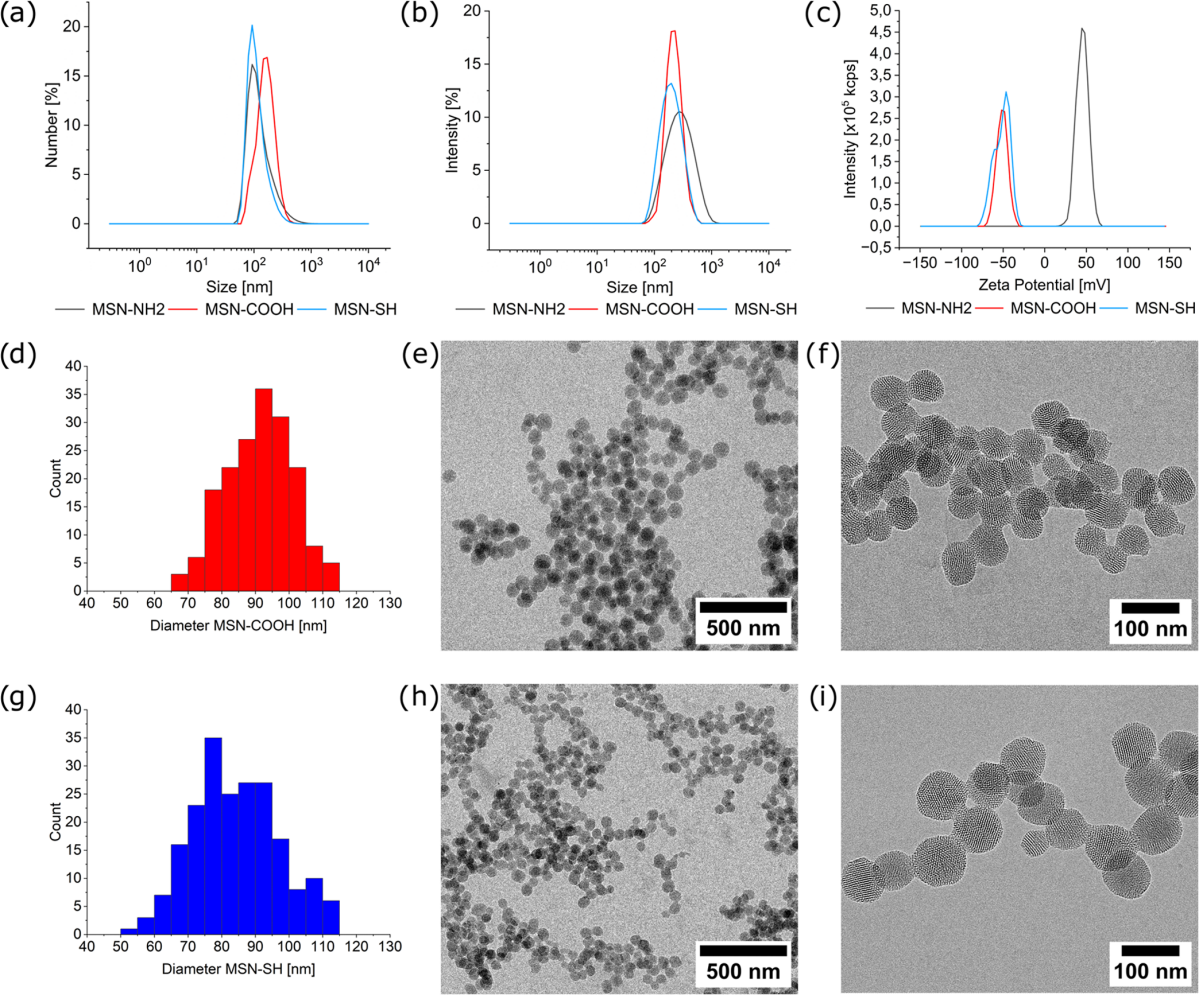
(a) DLS number-based particle size distributions, (b) DLS intensity-based particle size distribution, and (c) ζ-potential measurements of MSN-NH_2_ (black), MSN-COOH (red), and MSN-SH (blue). (d–f) Particle size distributions and representative transmission electron microscopy (TEM) micrographs of MSN-COOH and (g–i) particle size distributions and representative TEM micrographs of MSN-SH.

Functionalized MSNs were synthesized following a well-established co-condensation approach (MSN-SH, MSN-NH_2_) or postsynthetic functionalization route (MSN-COOH) as described in the Experimental Section. Dynamic light scattering (DLS) measurements of particle dispersions in water show a relatively narrow size distribution and small polydispersity indices (PI) for all three types of nanoparticles, indicating good dispersibility and colloidal stability of the MSN in aqueous solutions. The number-based hydrodynamic diameter was 160.0 ± 46.4 nm (*Z*-average: 185.4 nm, PI: 0.05) for MSN-NH_2_, 191.7 ± 60.4 nm (*Z*-average: 240.1 nm, PI: 0.21) for MSN-COOH, and 113.7 ± 37.8 nm (*Z*-average: 162.2 nm; PI: 0.2) for MSN-SH (Figure 2a,b).

The morphology, shape, and size of the mesoporous silica nanoparticles were further studied by transmission electron microscopy (TEM; Figures 2d–i and S1). These images show that the nanoparticles are monodisperse with a spherical shape and a mean particle size of 91.5 ± 9.9 nm for MSN-NH_2_ and MSN-COOH (which are based on the same batch of particles) and 84.3 ± 12.5 nm for MSN-SH. Both are smaller than the hydrodynamic diameters measured in DLS, which is normal due to hydrodynamic diameters including a solvent shell around the particles which is not observed in TEM, and DLS measuring an intensity-based particle size distribution as an equivalent sphere diameter (which can be converted into a number-based distribution following certain assumptions), while TEM directly gives a number-based particle size distribution. Furthermore, the conditions during TEM image acquisition (high vacuum and a high energy electron beam) can lead to nanoparticle shrinkage. Lastly, the difference in size can be an indication of the presence of (small) agglomerates or aggregates in solution.

The mesoporous structure that allows the particles to be loaded with metal ions is clearly observed in these images and further confirmed by N_2_ sorption measurements.

**Figure 3:**
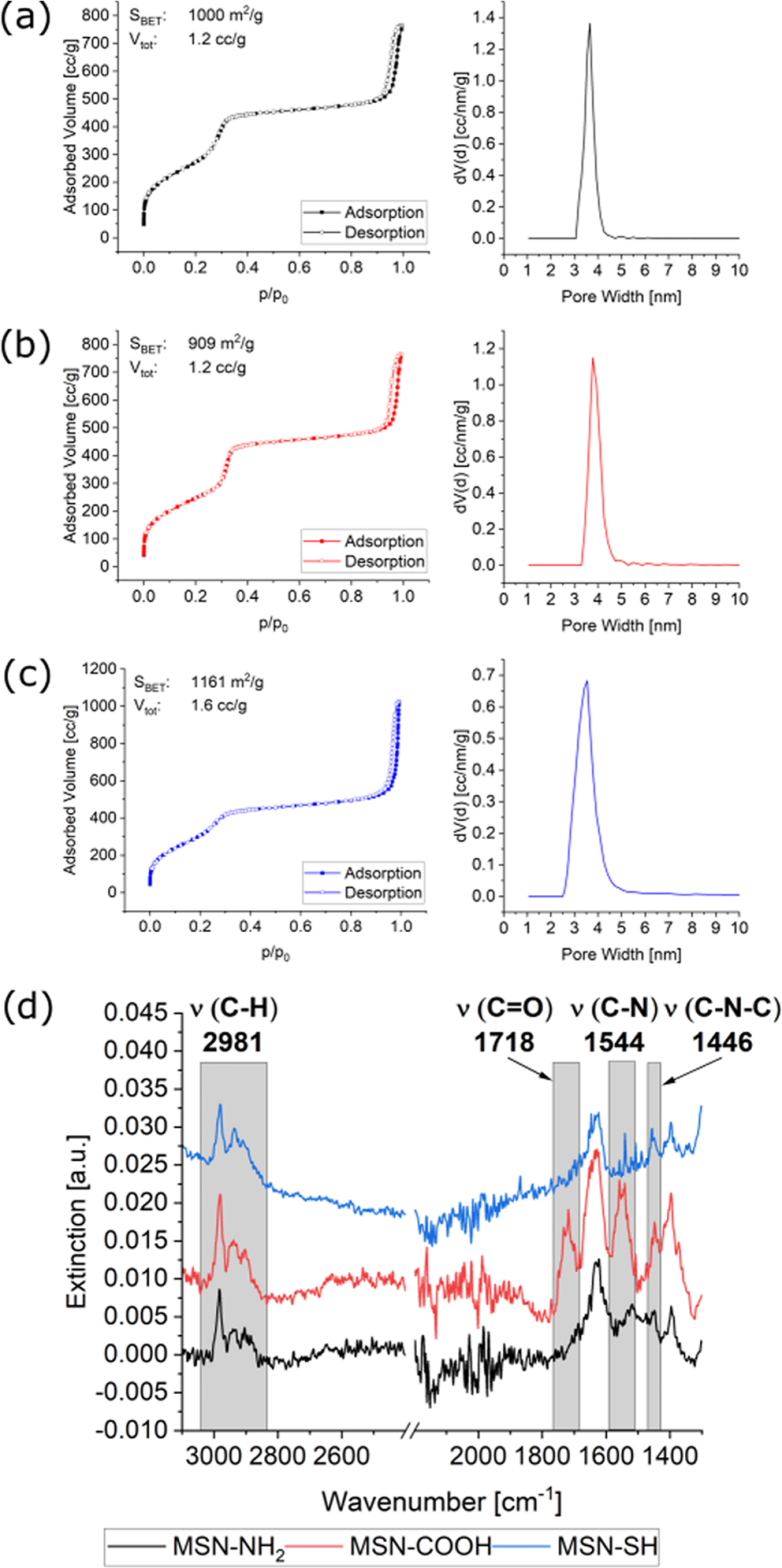
(a–c) Nitrogen sorption isotherms and NLDFT pore size distributions of MSN-NH_2_ (a), MSN-COOH (b), and MSN-SH (c). (d) Attenuated total reflection Fourier transform infrared spectroscopy (ATR-FTIR) spectra of MSN-NH_2_ (black), MSN-COOH (red), and MSN-SH (blue). An axis break was inserted in the region of atmospheric CO_2_ absorption (2200–2400 cm^–1^) for better clarity.

Nitrogen Sorption measurements show typical type IV isotherms, indicative of a mesoporous material (Figure 3a–c). The total BET surface area and total and mesopore pore volumes of the samples were calculated as 1000 m^2^/g, 1.2 cm^3^/g, and 0.7 cm^3^/g for MSN-NH_2_, 909 m^2^/g, 1.2 cm^3^/g, and 0.7 cm^3^/g for MSN-COOH, and 1161 m^2^/g, 1.6 cm^3^/g, and 0.7 cm^3^/g for MSN-SH. The pore diameters calculated with the Barrett–Joyner–Halenda (BJH) method and a nonlocal density functional theory (NLDFT) model were 3.0 and 3.5 nm for MSN-NH2, 3.0 and 3.8 nm for MSN-COOH, and 3.0 and 3.5 nm for MSN-SH, showing sharp and well-defined peaks from ordered mesopores, which is in line with the observations from the TEM images. These results further indicate a highly porous material with ample space inside the mesopores to carry and store antimicrobial metal ions. More porosity data can also be found in Table S1 in the Supporting Information.

ζ-Potential measurements of aqueous suspensions of the three differently functionalized MSNs were carried out to investigate their successful functionalization (Figure 2c). Typically, unfunctionalized MSNs exhibit a negative surface potential in neutral water due to the presence of partially deprotonated surface silanol groups. After functionalization with amine groups, MSN-NH_2_ showed a strongly positive ζ-potential of around +45 mV, due to the protonation of the amine groups at pH ∼ 7.^24^ As expected, the further functionalization of MSN-NH_2_ to yield MSN-COOH results in a pronounced shift of the ζ-potential from +45 to −47 mV due to the deprotonation of the carboxyl groups on the surface of MSN-COOH at neutral pH. MSN-SH also showed a negative ζ-potential of ca. −48 mV. These results indicate that the functionalization of the nanoparticles was successful and explain the good colloidal stability of the MSNs observed in DLS due to electrostatic stabilization.

Additionally, the successful functionalization of the MSN was further investigated by attenuated total reflection Fourier transform infrared spectroscopy (ATR-FTIR; Figure 3d).^25^ All silica nanoparticles exhibit strong Si–O and Si–O–H stretching and bending vibration bands in the range 400–1300 cm^–1^. MSNs functionalized with organic trialkoxysilanes also show the asymmetric stretching of C–H at around 2980 cm^–1^.^26,27^ Additionally, MSN-NH_2_ show a band at 1446 cm^–1^ from N–H vibrations.^28^ After the amine groups of MSN-NH_2_ were reacted with succinic anhydride to yield MSN-COOH through amide bond formation, the presence of new bands can be observed. The amide I band located at around 1718 cm^–1^ is mainly due to the C=O stretching vibrations, and the amide II band arising from C–N stretching vibrations can be seen at around 1544 cm^–1^.^29,30^ There is also a new band at 1446 cm^–1^ that can be assigned to the C–N–C vibration of the newly formed amide bond. These results indicate a successful functionalization of MSN-NH_2_ to MSN-COOH.

### 2.2 Investigation of Ion Loading and Release from MSN

**Figure 4:**
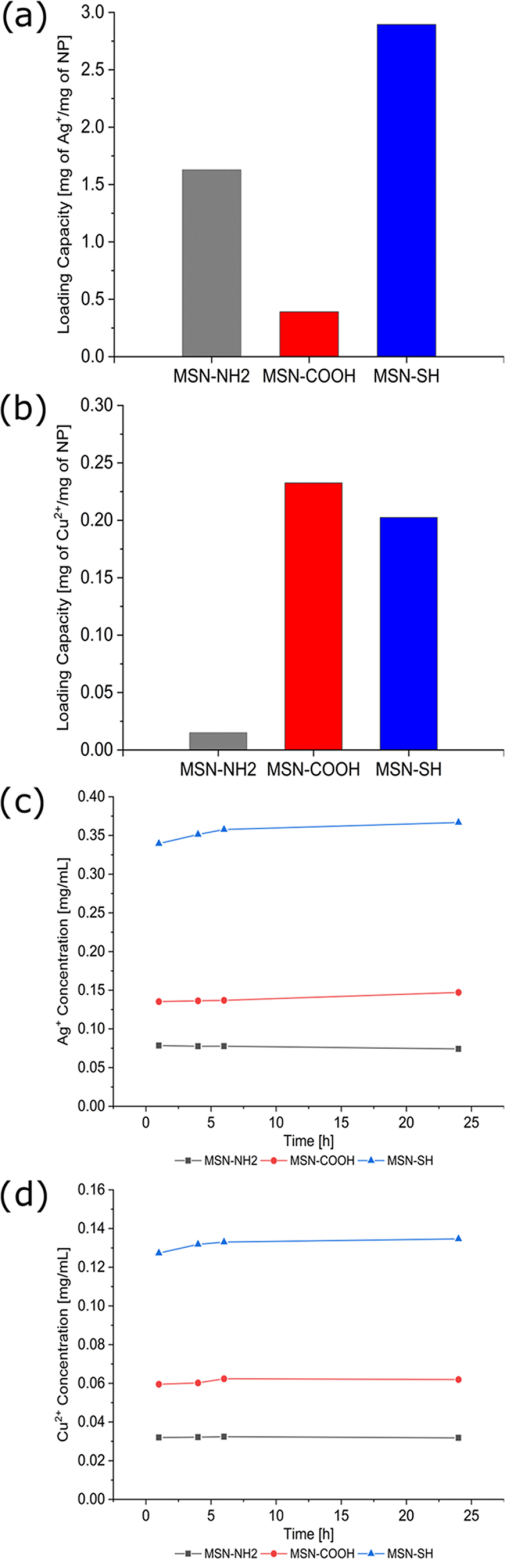
Loading capacities, in milligram of metal ions per mg of particles, for Ag^+^ (a) and Cu^2+^ (b), and concentration of released Ag^+^ (c) and Cu^2+^ (d) in Milli-Q water over time for 3 mg of MSN-NH_2_ (black), MSN-COOH (red), and MSN-SH (blue).

Depending on the interaction strength of the surface functional group on the nanoparticles with the metal ions, we expect a trade-off between the amount of ions that can be loaded into the nanoparticles and the amount that can be mobilized again from the pores and partitioned to the surrounding medium. A strong interaction between the metal ions and the functional groups usually results in a high loading capacity; however, if the interactions are too strong, the ions will stay bound to the inner pore walls of the nanoparticles and not be available to unfold their antimicrobial potential. Moreover, the release will depend on the protonation and deprotonation states of the functional groups during loading and release. To get a better understanding of these effects, we investigated the amount of copper and silver ions that can be loaded into the nanoparticles and that subsequently partition into the surrounding medium in Milli-Q water with inductively coupled plasma optical emission spectroscopy (ICP-OES).^31^Figure 4 shows the amount of both copper and silver ions that can be loaded into the differently functionalized particles, as well as their release in water over time (1, 4, 6, and 24 h). As expected, the functional groups on the nanoparticle surfaces influence the interaction with copper and silver ions during loading and release to different degrees. The loading capacities, expressed in milligrams of metal ions per mg of particles, were 0.39 mg/mg (MSN-NH_2_), 1.63 mg/mg (MSN-COOH), and 2.90 mg/mg (MSN-SH) for silver and 0.02 mg/mg (MSN-NH_2_), 0.23 mg/mg (MSN-COOH), and 0.20 mg/mg (MSN-SH) for copper (see Figure 4a,b). The total amount of released ions decreases in the order MSN-SH ≫ MSN-COOH > MSN-NH_2_. The relative release capacities under the tested conditions after 24 h were 1.5% for MSN-NH_2_, 12.6% for MSN-COOH, and 4.2% for MSN-SH for silver-loaded particles, and 70.8% for MSN-NH_2_, 8.9% for MSN-COOH, and 22.2% for MSN-SH for copper-loaded particles. This demonstrates the above-mentioned trade-off between the relative loading and release capacities and the total ion release in dependence of the particle functionalization. These results are also in line with the observation of the more application-relevant touch tests under semidry conditions, and the repeated touch tests in which the same surface was repeatedly exposed to new bacteria, as discussed in Section 2.4.

### 2.3 Deposition of Adhesion Layers and MSNs as Antimicrobial Surface Coatings

For testing the antimicrobial properties of the MSNs and to prove their effectiveness after deposition as a thin film on a surface, we produced antimicrobial surface coatings on 1 cm × 1 cm stainless steel substrates via a spray-coating technique. Stainless steel was used as a substrate due to its ubiquitous use in high-touch surfaces in hospitals, healthcare facilities, and nursing homes. To improve the wettability of the stainless steel surfaces and the adhesion of the particles to the substrates, we first coated the substrates with polyelectrolyte adhesion layers. We chose poly(allylamine hydrochloride) (PAH), poly(styrene sulfonic acid) sodium salt (PSS), and PAH functionalized with 2-iminothiolane (Traut’s reagent) at different molar ratios as adhesion layers, based on their ability to adhere well to stainless steel surfaces and strongly bind the differently functionalized nanoparticles through electrostatic or covalent interactions.

PAH is a widely used and commercially available polycationic molecule containing a large number of amine groups that are partially protonated at neutral pH.^31^ Stainless steel surfaces, on the other hand, exhibit a negative surface potential due to the presence of metal oxides and hydroxides. Consequently, we expect strong electrostatic interactions between PAH and the stainless steel surfaces. Furthermore, hydrogen bonding between the amine groups of PAH and surface OH groups is also expected to occur. Lastly, the lone pair of deprotonated amine groups can bind to metal atoms on the stainless steel surface coordinatively. This should result in a very strong adhesion of such polycations to stainless steel surfaces, which is also known from their use as corrosion inhibitors.^32,33^ Furthermore, the ionic nature and strong hydrophilicity of these layers improve the wettability of the surfaces and hence the homogeneity of the coatings with nanoparticle dispersions that are deposited in a subsequent step.

The adhesion layers not only need to adhere very well to the surfaces and facilitate the homogeneous deposition of the antimicrobially active nanoparticles as thin films but also need to strongly bind them to the substrates as well. Polycationic PAH is already expected to bind strongly to negatively charged MSN-COOH via electrostatic interactions and hydrogen bonding. For positively charged MSN-NH_2_, we introduced a second adhesion layer of PSS, a strongly negatively charged polyanion that adheres well to polycationic layers (as demonstrated by its frequent use together with PAH in layer-by-layer (LbL) approaches^34^) and can at the same time bind positively charged MSN-NH_2_. For MSN-SH, we investigated the possibility of binding the particles covalently to the adhesion layers via disulfide bonds after partially functionalizing some of the primary amine groups of PAH with 2-iminothiolane (Traut’s reagent) to yield thiol groups (referred to as PAH:TRAUT in this manuscript). These can readily react with free thiols on the surface of MSN-SH in the presence of oxygen from the air to form covalent disulfide bonds. The process is sped up even further in the presence of heavy metal ions such as silver and copper.^35^ Moreover, the positive charge of the amine groups is conserved after the reaction with 2-iminothiolane and the formation of the amidine, resulting in further electrostatic attraction between PAH:TRAUT and negatively charged MSN-SH. We investigated different ratios of PAH to Traut’s reagent (resulting in different degrees of functionalization and different amounts of thiol groups), because we expected a trade-off between the ability of thiolated PAH to adhere to stainless steel surfaces on the one hand and to covalently bind MSN-SH on the other hand.

**Figure 5:**
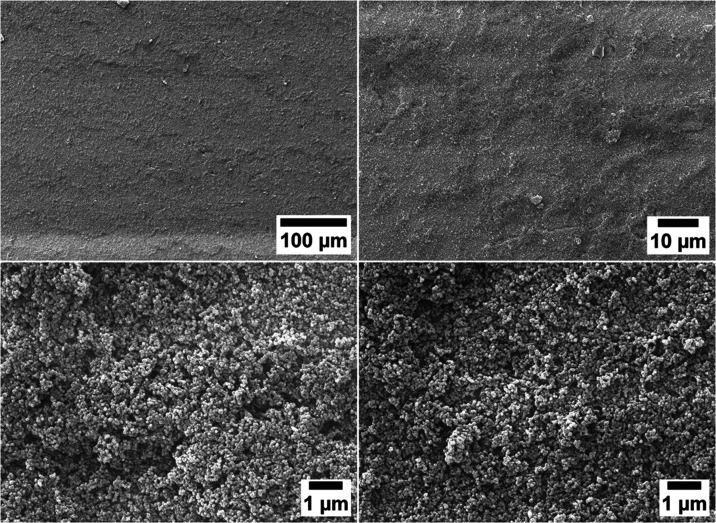
eSEM images at different magnifications and different locations of MSN-SH spray-coated on a PAH adhesion layer on stainless steel substrates, showing complete and homogeneous coverage of the substrate.

The successful deposition of the nanoparticles and the homogeneity of the spray-coated thin films on top of the adhesion layers on the stainless steel substrates were demonstrated with environmental scanning electron microscopy (eSEM). Figure 5 shows an overview image demonstrating the homogeneity of the coating over large ranges as well as two images at higher magnification from the center and edge of the substrate, showing the individual nanoparticles making up the surface coatings after depositing MSN-SH (2 mg/mL) on a PAH adhesion layer (spray-coated from an aqueous solution at a neutral pH and 5 mg/mL) onto a stainless steel surface.

Table 1 lists the various combinations of MSNs, metal ions, and adhesion layers that were produced and tested for their antimicrobial efficacy in this study based on the considerations explained above.

**Table 1:** Various MSNs Functionalized with Silver or Copper and Spray-Coated on Different Types of Adhesion Layers

MSN type	adhesion layer	metal ion
MSN-NH_2_	PAH (5 mg/mL)	Ag^+^
	PSS (5 mg/mL)	Cu^2+^
MSN-COOH	PAH (5 mg/mL)	Ag^+^
		Cu^2+^
MSN-SH	PAH (5 mg/mL)	Ag^+^
		Cu^2+^
	PAH:TRAUT (1:1)	Ag^+^
	PAH:TRAUT (2:1)	Ag^+^
		Cu^2+^
	PAH:TRAUT (6.7:1)	Ag^+^

To further characterize the thin films and their surface properties, we also determined the water contact angle of silver-ion-loaded coatings (see also Figure S4 in the Supporting Information). As expected, all coatings were very hydrophilic with water contact angles of 14.8 ± 3.7° for MSN-COOH/Ag, 4.5 ± 7.9° for MSN-SH/Ag, and 5.5 ± 1.4° for MSN-NH_2_/Ag. For comparison, uncoated stainless steel showed a water contact angle of 86.4 ± 2.8°. Even though the mode of action of the coatings discussed here is based on metal ion mobilization under (semi)dry conditions rather than on preventing microbial adhesion, the hydrophobicity of surfaces plays, among many factors, a key role in the attachment of microbes on surfaces. Hydrophobic surfaces tend to attract more hydrophobic bacteria, especially Gram-negative bacteria with hydrophobic cell wall such as *P. aeruginosa* (WCA of bacterial lawn reported to be approximately 80–130°), whereas hydrophilic surfaces are more prone to hydrophilic bacterial attachment, such as Gram-positive *S. aureus* (WCA 20–36°).^36^

The water contact angle of MSN-SH/Ag with the highest antimicrobial activity is close to superhydrophilicity (WCA 5.5 ± 1.4°), which might effectively repel hydrophobic Gram-negative pathogens. A more in-depth evaluation of microbial attachment on the developed coating to achieve not only a high killing effect by metal ions but also reduced microbial attachment for providing the highest protection for high-touch surfaces is beyond the scope of the current study. However, tuning the composition of the coating, especially the adhesive layer, could be further optimized to achieve desirable surface wettability.

Depth profiling of a scratched film of MSN-SH on PAH:Traut (2:1) revealed a film thickness of 1.5 μm. The mass of the deposited coatings for MSN-SH on PAH:Traut (2:1) loaded with silver and copper were determined as 115 μg/cm^2^ and 74 μg/cm^2^, respectively.

### 2.4 Antimicrobial Activity

**Figure 6:**
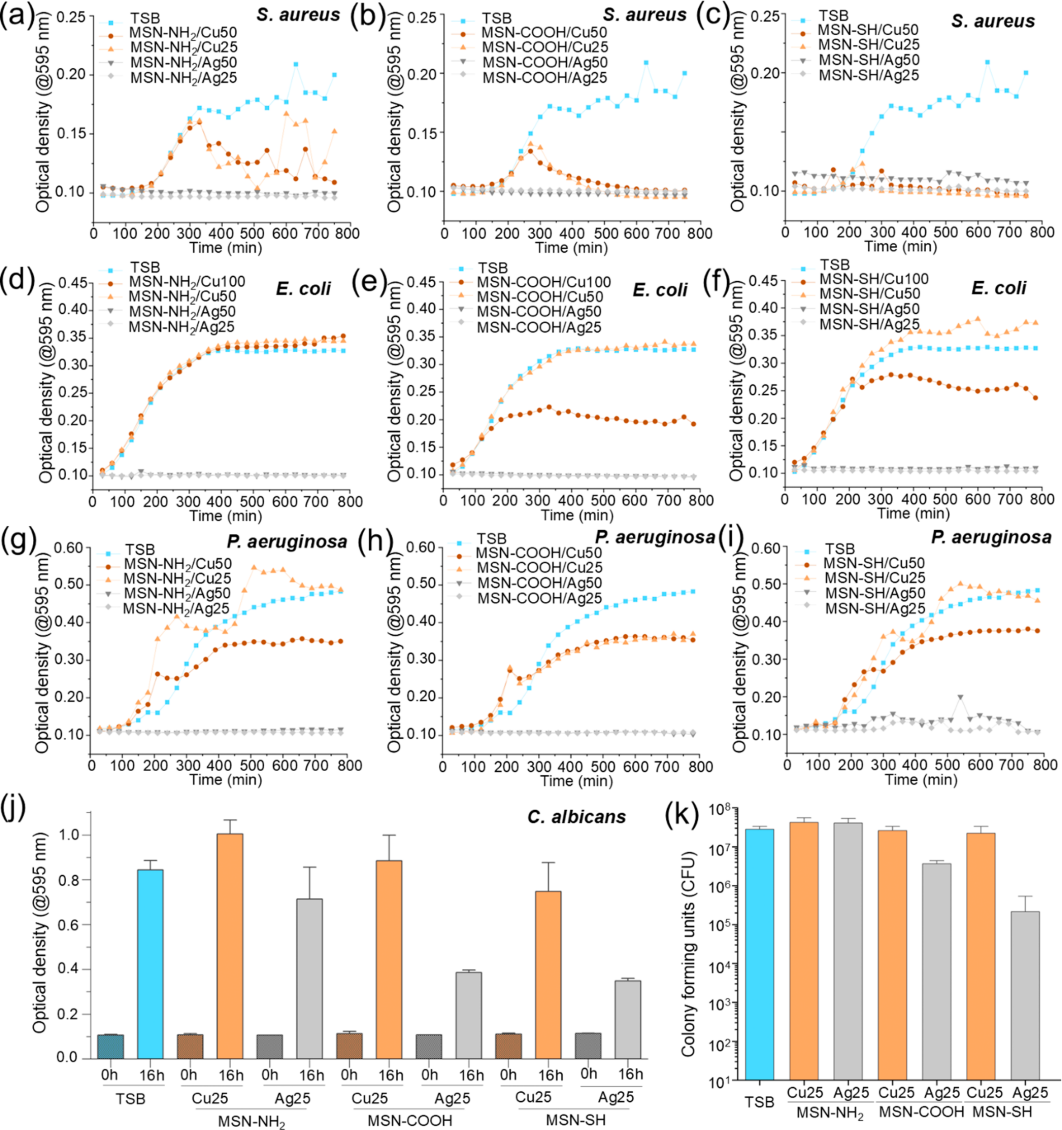
Antimicrobial activity of MSN in a solution. (a–c) Growth (optical density) of *S. aureus* cultured in media without nanoparticles (TSB) or with MSN-NH_2_ (a), MSN-COOH (b), or MSN-SH (c) at 25 or 50 μg/mL. (c). (d–f) Growth of *E. coli* in media without nanoparticles or with MSN-NH_2_ (d), MSN-COOH (e), or MSN-SH (f) at 50 or 100 μg/mL for Cu-loaded MSN and 25 or 50 μg/mL for Ag-loaded MSN. (g–i) Growth of *P. aeruginosa* in media without nanoparticles or with MSN-NH_2_ (g), MSN-COOH (h), or MSN-SH (i) at 25 or 50 μg/mL. (j) Optical density of *C. albicans* culture at incubation time 0 and 16 h incubated without nanoparticles or with MSN at 25 μg/mL. (k) Colony-forming units of *C. albicans* incubated without nanoparticles or with MSN at 25 μg/mL.

The antimicrobial activity of the nanoparticles was first assessed by monitoring the growth of four representative pathogenic microbes in the presence of nanoparticles: a Gram-positive bacterium (*S. aureus*, Figure 6a–c), two Gram-negative bacteria (*E. coli* and *P. aeruginosa*, Figure 6d–f,g–i, respectively), and a fungus (*C. albicans*, Figure 6j,k). For the bacterial strains, growth was quantified by measuring the optical density (OD) at 595 nm of the culture suspension, in both the presence and absence of nanoparticles. Due to the formation of inhomogeneous macro- and microcolonies in *C. albicans* culture, OD measurements were only taken at the start and end of the culture period, as intermediate readings were unreliable. Instead, the colony-forming units (CFUs) were quantified at the end point using the plate pour method. Nanoparticle concentrations of 25 and 50 μg/mL were used for all microbes, as higher concentrations interfered with OD measurements.

All nanoparticles loaded with silver ions effectively inhibited the growth of both Gram-positive and Gram-negative bacteria when added to the culture at a concentration of 25 μg/mL or higher. For *C. albicans*, the inhibition was less efficient, and the antimicrobial effect varied depending on the type of MSN. MSN-SH was found to be the most efficient, showing approximately 2log_10_ reduction of CFU compared to the control without nanoparticles. (Figure 6k) The observed minimal antimicrobial efficacy of the nanoparticles for the fungus aligns with previous findings, where *C. albicans* exhibited the highest minimum inhibitory concentration (MIC) and minimum bactericidal concentration (MBC) for silver nanoparticles compared to *E. coli* and *S. aureus*.^37^ The lower susceptibility to metal nanoparticles of *C. albicans* is possibly attributed to the different structure and composition of fungal cell walls from bacterial cell membranes, where the fungal cell wall thickness is around 0.1–1 μm and mainly composed of polysaccharides,^38^ whereas bacterial cell membranes are typically below 100 nm thick.^39^ Copper-loaded nanoparticles generally exhibited limited antimicrobial efficacy compared with silver MSNs. This can be partially caused by the lower availability of copper ions for microbes (Figure 4). For *S. aureus*, the particles inhibited growth relatively efficiently, with MSN-SH/Cu demonstrating the highest efficacy, followed by MSN-COOH and MSN-NH_2_. However, these particles did not show significant inhibitory effects against the two Gram-negative bacteria or the fungus. Copper ions are generally more effective against Gram-positive bacteria than Gram-negative bacteria because they bind to amine and carboxyl groups on the surface of Gram-positive bacteria.^40^ Our results are consistent with this observation.

**Figure 7:**
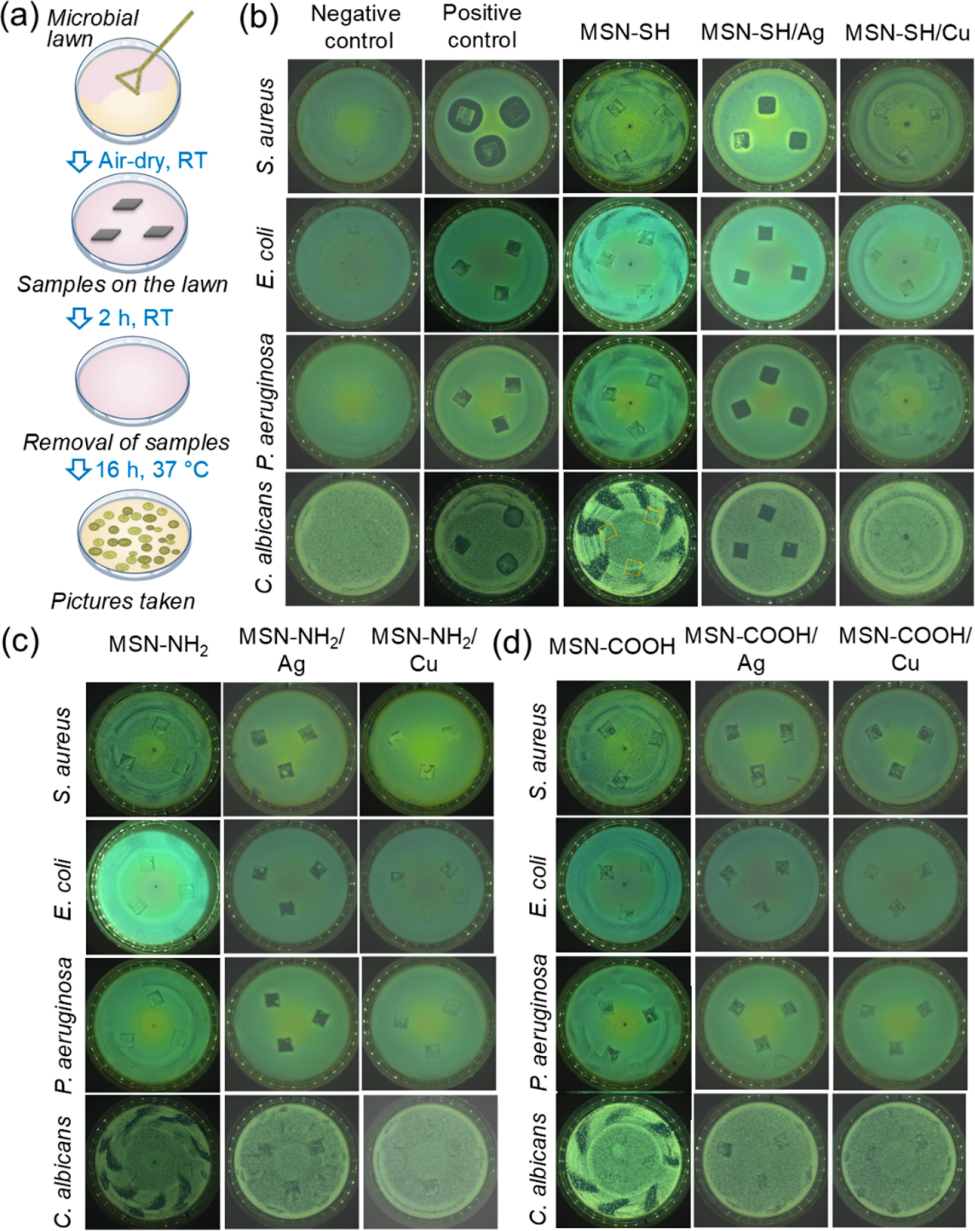
Antimicrobial activities of nanoparticles coated on stainless steel. (a) Schematic of touch test. (b–d) Growth of microbial colonies after being touched by each sample: (b) MSN-SH unloaded, loaded with Ag or Cu on PAH:TRAUT 2:1 adhesion layers; (c) MSN-NH2 unloaded, loaded with Ag or Cu on PAH/PSS adhesion layers; and (d) MSN-COOH unloaded, loaded with Ag or Cu on PAH adhesion layers. Unmodified stainless steel was used as a negative control. Quaternary ammonium-based antimicrobial coating on stainless steel was used as a positive control.

Next, the antimicrobial efficacy of the nanoparticle coatings was assessed. To simulate the semidry environment of finger skin touching a coating, we employed an in-house method called the Touch test. In this test, sample substrates are placed in triplicate on the surface of a microbial lawn on an agar plate (Figure 7a). When the substrate is not antimicrobially active, the growth of microbial colonies in contact with the substrate is unaffected, resulting in full coverage of colonies (Figure 7b, positive control). For antimicrobial substrates, microbial growth is inhibited, leading to the formation of a colony-free zone on the agar gel (Figure 7b, negative control). Moreover, control samples with just the adhesion layers and unloaded MSN-SH, MSN-NH_2_, and MSN-COOH particles coated on stainless steel substrates showed no antimicrobial activity, proving the general biocompatibility of the MSN and the fact that the antimicrobial activity is due only to the metal ion payload and not the particles or adhesion layers themselves (Figures 7 and S2).

In general, the antimicrobial efficacy of the nanoparticle coatings showed a trend similar to those of nanoparticle suspensions. MSN-SH loaded with Ag showed the highest efficacy against all four strains (Figure 7b), which was even comparable to the positive control, prepared by spray-coating of a quaternary ammonium-based commercial product. Interestingly, in the Touch test, the growth of *C. albicans* was more efficiently inhibited by the MSN-SH/Ag coating compared to the liquid test. One possible reason underlying this observation is the faster growth of microbes in liquid culture compared to solid agar plates, due to better nutrient circulation^41^ or higher local concentration of antimicrobials on the given surface. MSN-NH_2_/Ag and MSN-COOH/Ag also demonstrated a relatively efficient inhibition of microbial growth (Figure 7c,d). All three types of MSN with copper ions did not significantly affect microbial growth on the agar plate. While they exhibited certain antimicrobial activity against *S. aureus* in the liquid test, their antimicrobial effect was very limited in the touch test. This could be due to the semidry conditions on the agar plate, where the mobilization of ions is less efficient than in liquid environments, thereby reducing the effective ion concentration available to bacteria.

Overall, the observed highest antimicrobial efficacy of MSN-SH-based materials suggests that these nanoparticles can carry large amounts of metal ions due to very favorable thiol-Ag/Cu interactions. However, these interactions are not so strong as to completely inhibit the release or mobility of the ions. Another possible reason for the observed high antimicrobial efficacy of MSN-SH is that MSN-SH can interact with surface-exposed proteins via disulfide bond formation with cysteine residues.^42−44^ If this is the case, the local ion concentration near cells would be higher compared to scenarios with fewer interactions between the particles and cells. The exact mechanisms underlying the high antimicrobial efficacy of MSN-SH-based materials need to be thoroughly investigated in a follow-up study.

To assess the mechanical stability of the coating, we conducted abrasion tests, where a sandpaper holder was used for scrubbing the samples, followed by antimicrobial touch testing (Figure S5). Here, we also included an uncoated and untreated copper surface as a positive control for comparison. Additionally, we performed a repeated touch test, where the same coating was exposed to a fresh bacterial lawn three times, each for a duration of 2 h (Figure S6). Both tests qualitatively confirmed that the coatings exhibit significantly higher antimicrobial properties compared with stainless steel, even after simulated wear and repeated touches. However, the antimicrobial activity was lower than that of coatings not subjected to mechanical stress or repeated touches. A more detailed quantitative assessment of the mechanical stability of the coatings will be conducted using an abrasion test under conditions mimicking real-life settings in a follow-up study.

## 3 Conclusions

We synthesized mesoporous silica nanoparticles with three different surface functional groups: MSN-NH_2_, MSN-COOH, and MSN-SH. Characterization with TEM and DLS revealed spherical, monodisperse particles around 80–100 nm. The successful functionalization of the nanoparticles was further proved with ATR-FTIR and ζ-potential measurements, and particle porosity was confirmed using TEM and Nitrogen Sorption measurements. The particles could be readily loaded with antimicrobially active silver and copper ions by impregnation in aqueous AgNO_3_ and CuSO_4_ solutions.

Before spray-coating these nanoparticle dispersions on stainless steel surfaces, we investigated different polyelectrolyte adhesion layers to improve the wettability of the surfaces and hence the homogeneity of the resulting coatings as well as the adhesion of the nanoparticles to the substrates. The polyelectrolytes were chosen to be complementary in charge and reactivity to the surface functional groups of the MSN.

After depositing the nanoparticles on stainless steel substrates primed with adhesion layers, we tested their antimicrobial efficacy against *E. coli*, *P. aeruginosa*, *S. aureus*, and *C. albicans*. The best results were obtained for silver-ion-loaded MSN-SH on PAH:TRAUT adhesion layers, which showed very high antimicrobial activity against all four tested pathogens.

In the future, we will further investigate the use of these coatings in real-life scenarios such as coatings on doorknobs in healthcare institutions and nursing homes. One key factor will be to test and ensure the absence of cytotoxicity of these coatings toward human skin cells. Moreover, in addition to the antibacterial and antifungal properties of the coatings that have already been demonstrated in this work, we will also investigate their antiviral properties. Lastly, we will investigate whether antimicrobial resistance can arise against these coatings and how these effects can be mitigated.

## 4 Experimental Section

### 4.1 Materials

Hexadecyltrimethylammoniumbromide (CTAB, purity ≥99%, ROTH), sodium hydroxide (1M, ChemSolute), tetraethyl orthosilicate (TEOS, purity ≥99%, Sigma-Aldrich), (3-aminopropyl)triethoxysilene (APTES, purity ≥98%, Sigma-Aldrich), 3-mercaptopropyltriethoxysilane (purity 97%, Fluorochem), hydrochloric acid (37.0%, ChemSolute), ethanol (Denatured, purity ≥99.8%, ChemSolute), *N*,*N*-dimethylformamide (DMF, purity ≥99.9%, Carlo Erba), succinic anhydride (purity 99%, Thermo Scientifc), triethylamine anhydrous (Fluorochem), ammonium nitrate (purity >98%, Chemlab), nitric acid (purity >65%, ChemSolute), poly(styrene sulfonic acid) sodium salt (PSS, *M*_w_ 70,000, Thermo Scientific), poly(allylamine hydrochloride) (PAH, ThermoScientific), and 2-iminothiolane hydrochloride (Traut’s reagent, purity ≥98%, Sigma-Aldrich), ICP standards (1.000 g Ag/L, 1.000 g Cu/L, Bernd Kraft), TSB Tryptic Soy Broth (100%, Sigma-Aldrich), and d-glucose anhydrous (Carl Roth) were used as received. If not mentioned otherwise, water refers to Milli-Q-filtered water (Milli-Q, Merck-MilliPore) in this manuscript. Stainless steel substrates (AISI 304 steel; DIN 1.4301–1.4307; 1 cm × 1 cm) with a bright annealed finish from cold rolling were obtained from ARST. Bacterial strains *S. aureus* (ATCC 6538P), *P. aeruginosa* (ATCC 15442), and *E. coli* (ATCC 8739), and a fungal strain *C. albicans* (DSM11224) were kindly donated by the Federal Institute for Materials Research and Testing (BAM, Berlin, Germany).

### 4.2 Characterization

Transmission electron microscopy (TEM) images were acquired on a Talos F200S Microscope (Thermo Fisher Scientific) using an accelerating voltage of 200 kV. The samples were prepared by placing ∼10 μL of a dispersion of the nanoparticles in ethanol on a carbon-coated copper grid (Plano GmbH, 400 mesh) and letting them dry at room temperature. The obtained micrographs were analyzed with the software ImageJ by manually measuring the maximum Feret diameter of at least 170 particles from 3 different images.

Environmental scanning electron microscopy (eSEM) images were acquired on an FEI XL30 eSEM using an accelerating voltage of 20 kV. Before measurement, a thin (30 nm) film of Gold was sputter-coated on the samples using an EM ACE600 (Leica) sputter coater to make them conductive.

Attenuated total reflection Fourier transform infrared spectroscopy (ATR-FTIR) spectra were collected on a Nicolet Nexus FTIR spectrometer (Thermo Electron Corporation) using a Golden Gate ATR accessory. The spectra were recorded in a wavenumber range of 4000–550 cm^–1^. A new background spectrum was acquired before each measurement and subtracted from the sample spectrum to obtain background-corrected IR spectra.

Dynamic light scattering (DLS) and electrophoretic light scattering (ζ-potential measurements) were performed on a Zetasizer Ultra Red (Malvern Panalytical) using Malvern DTS 1070 folded capillary Zeta cells. 1 mL of a dispersion of the nanoparticles (0.125 mg/mL) in Milli-Q water (pH 7) was introduced into the cuvette and equilibrated at 25 °C, 3 measurements with 10 runs (DLS) and 12 runs (ζ-Potential Measurements) each were performed, and the mean and standard deviations were calculated.

Inductively coupled plasma optical emission spectroscopy (ICP-OES) was measured on a 5800 ICP-OES instrument (Agilent Technologies). For calibration, 8 standard solutions of Ag^+^ or Cu^2+^ in the concentration range of 50 ppm to 10 ppb were prepared in diluted HNO_3_ (0.066 mol/L). For quantification, the Ag emission line at 328.068 nm and the Cu emission line at 327.395 nm were used. The loading capacities of silver and copper ions were determined by dispersing 3 mg of metal-ion-loaded nanoparticles in 1 mL of 35% nitric acid and sonicating the mixture for 5 min for particle digestion. A 100 μL aliquot was then diluted with 9900 μL of Milli-Q water. Subsequently, 1 mL of this solution was further diluted in 9 mL of 66 mM HNO_3_, and ICP-OES was measured. The released amounts of ions at different time points were measured by dispersing 3 mg of metal-ion-loaded nanoparticles in 1 mL of Milli-Q water. Four separate solutions were prepared for each type of nanoparticle and metal ion loading in parallel, and after 1, 4, 6, and 24 h, one of these four solutions was centrifuged (7 min at 18k rcf), 800 μL of the supernatant were taken off, diluted in 9200 μL of diluted HNO_3_ (66 mM), and stored in the dark before measuring ICP-OES.

Nitrogen sorption measurements were performed on an ASAP 2020 (Micromeritics) instrument at liquid nitrogen temperature (77 K). Approximately 25 mg of sample was degassed for 16 h at 120 °C under vacuum. BJH and NLDFT pore size distributions were calculated from the desorption branch of the isotherm with the software Quantachrome ASiQwin v 3.01, using an equilibrium model for N_2_ on silica with cylindrical pores for NLDFT. BET surface areas were calculated from the linear part of the BET plots in a partial pressure range from ∼0.03 to ∼0.23, giving correlation coefficients of at least 0.999. The mesopore volume was calculated from the NLDFT model for pores smaller than 6.5 nm in diameter.

The water contact angle (WCA) was determined using an optical contact angle measuring and contour analysis system (Dataphysics) with a humidity controller set to 40% relative humidity. A 2 μL water droplet was placed on the surface, and a photo was taken after 5 s to measure the WCA. For each sample, the measurement was conducted in triplicate.

The thickness of the MSN-SH on the PAH:Traut (2:1) coating was determined by depth profiling of a scratched film with a Micromaterial NanoTest system (Micro Materials Ltd., Wrexham, U.K.).

The mass of the deposited coatings for MSN-SH on PAH:Traut (2:1) loaded with silver and copper were determined by scraping off the films from five 2.5 × 2.5 cm^2^ substrates with a razor blade, combining the material, and weighing it on an ultramicrobalance (Mettler Toledo XP2U).

### 4.3 Synthesis of Functionalized MSN (MSN-SH, MSN-NH_2_, MSN-COOH)

Mesoporous Silica Nanoparticles with different functional groups were synthesized according to a published procedure with modifications.^45^ In brief, 200 mg of CTAB and 1200 μL of sodium hydroxide solution (1.000 M) were dissolved in 100 mL of water under stirring. The solution was heated at 80 °C for 30 min, followed by the addition of a mixture of 1000 μL of TEOS and 10 mol % of the respective functionalized trialkoxysilane (i.e., (3-mercaptopropyl)triethoxysilane for mesoporous silica nanoparticles functionalized with thiol groups (MSN-SH) or (3-aminopropyl)triethoxysilane for mesoporous silica nanoparticles functionalized with amine groups (MSN-NH_2_)—which can be used for further functionalization to give mesoporous silica nanoparticles functionalized with carboxy groups (MSN-COOH)) under vigorous stirring. Stirring was continued for 2 h at 80 °C, and the solution was allowed to cool to room temperature. The nanoparticles were collected by centrifugation (12 min at 11k rcf), and washed 2× with water (2× 90 mL) and 2× with ethanol (2 × 90 mL).

To extract the organic template from the pores, the nanoparticles were dispersed in 90 mL of an ethanolic ammonium nitrate solution (1 g/50 mL), refluxed for 1 h, collected by centrifugation (12 min at 11k rcf), washed 1× with ethanol (90 mL), and redispersed in 90 mL of an acidic ethanolic solution (EtOH:HCl (conc.) = 90/10 (v/v)), refluxed again for 1 h, collected by centrifugation (12 min at 11k rcf), washed 2× with ethanol (2 × 90 mL) and stored in ethanol.

Carboxy-functionalized mesoporous silica nanoparticles (MSN-COOH) were synthesized from amine-functionalized mesoporous silica nanoparticles (MSN-NH2) following a published procedure.^46^ Briefly, 150 mg of amino-functionalized MSNs were added into 15 mL of DMF solution and redispersed. Then, a mixture of 1.0 g of succinic anhydride (10.0 mmol), 15 mL of DMF, and 255 μL of NEt_3_ (1.9 mmol) were added. The reaction mixture was stirred at room temperature for 24 h under an inert atmosphere of dry argon. The resulting product was collected by centrifugation (12 min at 11k rcf), washed with DMF and ethanol, and stored in ethanol.

### 4.4 Loading of MSNs with Metal Ions

MSNs (15 mg/mL) were loaded with silver ions or copper ions by incubating them in an aqueous solution of AgNO_3_ or CuSO_4_·5H_2_O (100 mg/mL) overnight at room temperature on a shaker. Afterward, the loaded MSNs were washed twice with Milli-Q water and redispersed in ethanol at a concentration of 2 mg/mL for spray-coating.

The procedure for loading the particles for ICP-OES measurements was the same as that for spray-coating.

### 4.5 Spray-Coating of Metal-Ion-Loaded MSN

Antimicrobial surface coatings were prepared on 1 cm × 1 cm stainless steel substrates via spray-coating from an airbrush with a nozzle size of 0.3 mm at a distance of 2 cm above the surface. Immediately before film deposition, the protective cover was removed from the substrates, they were cleaned with Ethanol, and then adhesion layers of PAH, PAH followed by PSS, or PAH functionalized with thiol groups through reaction with 2-iminothiolane hydrochloride (Traut’s reagent) were spray-coated on the substrates. The solutions were prepared as follows: PAH was dissolved in Milli-Q water at 5 mg/mL and brought to pH 7 with NaOH (1M). PSS was dissolved in Milli-Q water at 5 mg/mL and used directly. Thiol-functionalized PAH with different degrees of functionalization was prepared by dissolving PAH in Milli-Q water at 5 mg/mL, adding different amounts of Traut’s reagent (7.5 1.7, and 0.5 mg/mL, corresponding approximately to molar ratios of amine groups to Traut’s reagent of 1:1, 2:1 and 6.7:1, respectively), stirring the resulting solution for 2 h at room temperature, and then adjusting the pH to 7 with NaOH (1M). After drying, the antimicrobially active, metal-ion-loaded MSNs were spray-coated on the adhesion layers from an ethanolic solution at 2 mg/mL using the same air brushes and process parameters.

### 4.6 Antimicrobial Testing

To test the antimicrobial efficacy of each NP in a solution, the nanoparticle suspension in ethanol was redispersed in the Tryptic Soy Broth (TSB) media at predefined concentrations. Each microbial strain was precultured in 30% TSB supplemented with 0.25% glucose (TSB media) at 37 °C and 160 rpm overnight. Microbial suspensions with an optical density (OD) of 0.01 for the three bacteria and 0.05 for the fungus at 595 nm were prepared by diluting each preculture with TSB media. These suspensions were incubated at 37 °C and 160 rpm for 1.5 h to reach the exponential growth phase. Afterward, the OD value was adjusted to 0.02 for bacteria and 0.05 for fungi. The suspension was then transferred to 96-well plates at 50 μL per well. Subsequently, 50 μL of each nanoparticle suspension at different concentrations was added to each well, resulting in a final volume of 100 μL per well. The OD values of the plates were monitored every 30 min at 37 °C with shaking, using a microplate reader (BioTek Synergy H1 Plate Reader).

The antimicrobial efficacy of NP-coated surfaces was tested by Touch test, an in-house method for qualitative testing of antimicrobial surface under semidry conditions.^47^ For the touch test, all samples were sterilized by submerging them in 70% ethanol for 5 min, followed by air drying. Each microbial strain was precultured overnight in 30% TSB supplemented with 0.25% glucose (TSB media) at 37 °C and 160 rpm. Microbial suspensions with an OD of 0.1 at 595 nm were prepared by diluting each preculture with TSB media. These suspensions were incubated at 37 °C and 160 rpm for 1.5 h. Subsequently, the microbial suspension was diluted with 0.9% NaCl to obtain microbial suspensions with OD_600_ values of 0.1 for three bacterial strains and 0.5 for *C. albicans*. To prepare the microbial lawns, 100 μL of each diluted microbial suspension was uniformly spread on PC-agar plates using a spiral plater (Easyspiral pro, Interscience, Puycapel, France). The plates were allowed to dry fully in a biosafety cabinet for less than 20 min. Samples were placed upside down on the bacterial lawn in triplicate, ensuring the active surface was in contact with the microbes. The plates were then incubated at room temperature for 2 h. After incubation, the samples were carefully removed, and the plates were incubated overnight at 37 °C. The following day, images of the plates were taken using the Scan 300 colony counter (Interscience).

Wear resistance was assessed by touch abrasion and simulated using a BYK Garder scrub apparatus equipped with a dry microfiber cloth. The test employed a sandpaper holder with an approximate weight of 420 g, without any additional weight. The scrubbing was conducted at a frequency of 25 cycles per minute over a duration of 40 min, resulting in a total of 1000 cycles. Each cycle comprised two wipes and touches, covering a total path length of 40 cm. The sandpaper holder, with a surface area of 72.96 cm^2^, exerted a force of 56.47 N/cm^2^ (5.76 g/cm^2^) on the samples. This setup ensured consistent and reproducible abrasion conditions across all of the tested samples. The sample was then tested using the Touch test, as explained above, to confirm antimicrobial activity.

The antimicrobial activity upon repeated touches was qualitatively assessed in a repeated touch test according to the Touch test method outlined above. In this procedure, each sample was initially touched to a microbial plate. After each touch, the sample was transferred to a fresh microbial lawn. This process was repeated three times, with each touch involving a contact time of 2 h with the microbial plate. Afterward, the samples were removed, and the plates were incubated overnight at 37 °C. The following day, images of the plates were taken.

## Supporting Information Available

Particle morphology, antimicrobial activity, touch tests, water contact angle measurements, wear resistance, and additional nitrogen sorption data (PDF)

## Abbreviations

ATR-FTIR: attenuated total reflection Fourier transform infrared spectroscopy
CFUs: colony-forming units
DLS: dynamic light scattering
eSEM: environmental scanning electron microscopy
ICP-OES: inductively coupled plasma optical emission spectroscopy
MBC: minimum bactericidal concentration
MIC: minimum inhibitory concentration
MSN: mesoporous silica nanoparticle
MSN-NH2: mesoporous silica nanoparticles functionalized with amine groups
MSN-COOH: mesoporous silica nanoparticles functionalized with carboxy groups
MSN-SH: mesoporous silica nanoparticles functionalized with thiol groups
NP: nanoparticle
OD: optical density
TSB: Tryptic Soy Broth
PAH: poly(allylamine hydrochloride)
PSS: poly(styrene sulfonic acid) sodium salt
PAH:TRAUT: PAH functionalized with 2-iminothiolane (Traut’s reagent)
