# Supplementary material for: Facile Spray-Coating of Antimicrobial Silica Nanoparticles for High-Touch Surface Protection
Source: ACS Appl Mater Interfaces. 2025 Feb 12;17(8):12507–19. doi: 10.1021/acsami.4c18916 (PMC11873980; doi:10.1021/acsami.4c18916)
Supplement: Supplementary file 1 — am4c18916_si_001.pdf [file am4c18916_si_001.pdf]

Supporting Information

Facile Spray-Coating of Antimicrobial Silica Nanoparticles for High-Touch Surface Protection

Carolina Duarte Bernardino[†,§], *Mihyun Lee*[&,*], *Qun Ren*[&], *Bastian Ruehle*[†,*]

[†] Federal Institute for Materials Research and Testing (BAM), Richard-Willstätter-Strasse 11, D-12489 Berlin, Germany

[§] Humboldt University Berlin, Unter den Linden 6, D-10117 Berlin, Germany

[&] Laboratory for Biointerfaces, Empa, Swiss Federal Laboratories for Materials and Technology, Lerchenfeldstrasse 5, 9014 St. Gallen, Switzerland

* Correspondence: bastian.ruehle@bam.de, Mihyun.Lee@empa.ch

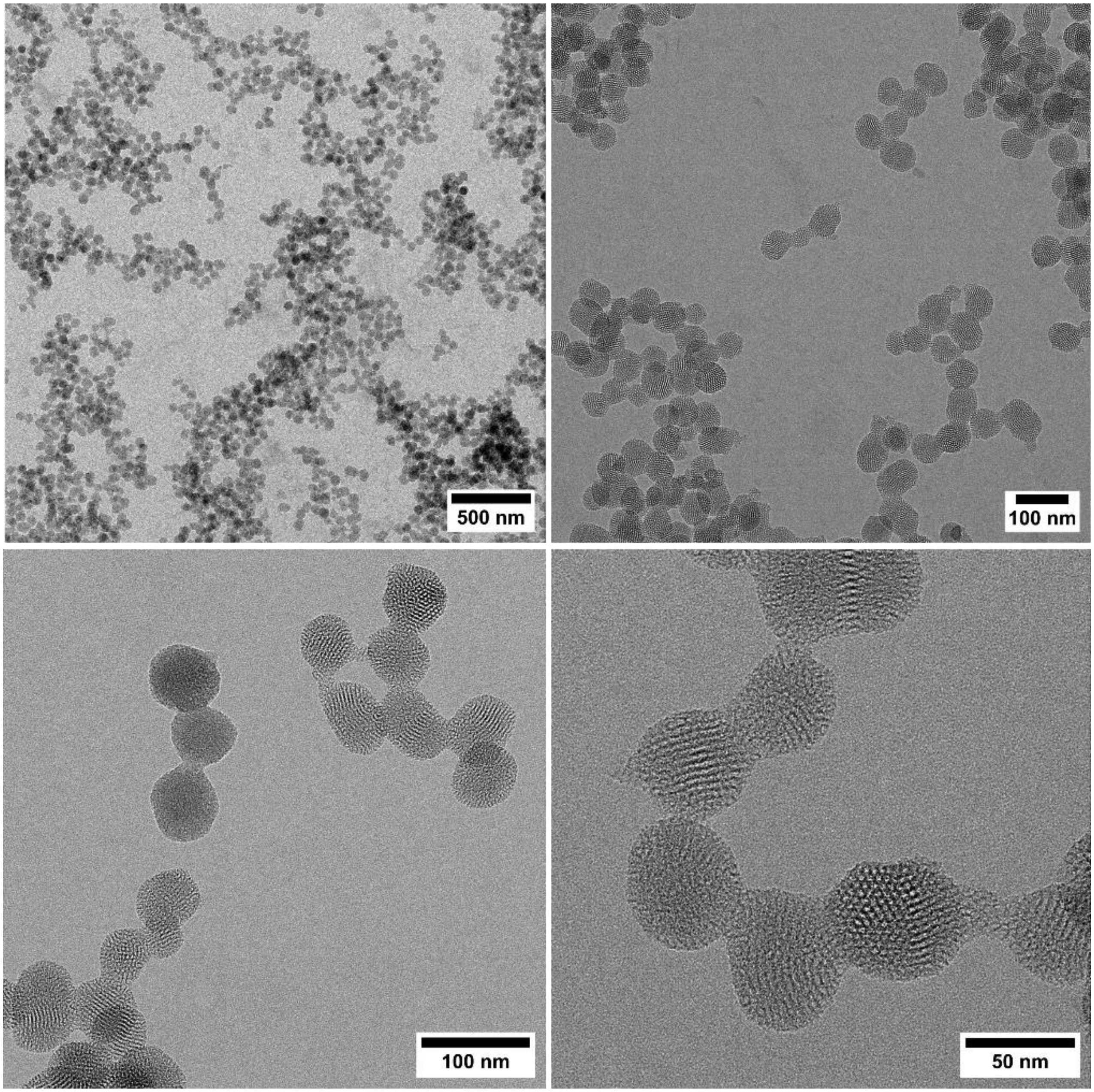

Figure S1 TEM images at different magnifications and different TEM grid locations of MSN-SH.

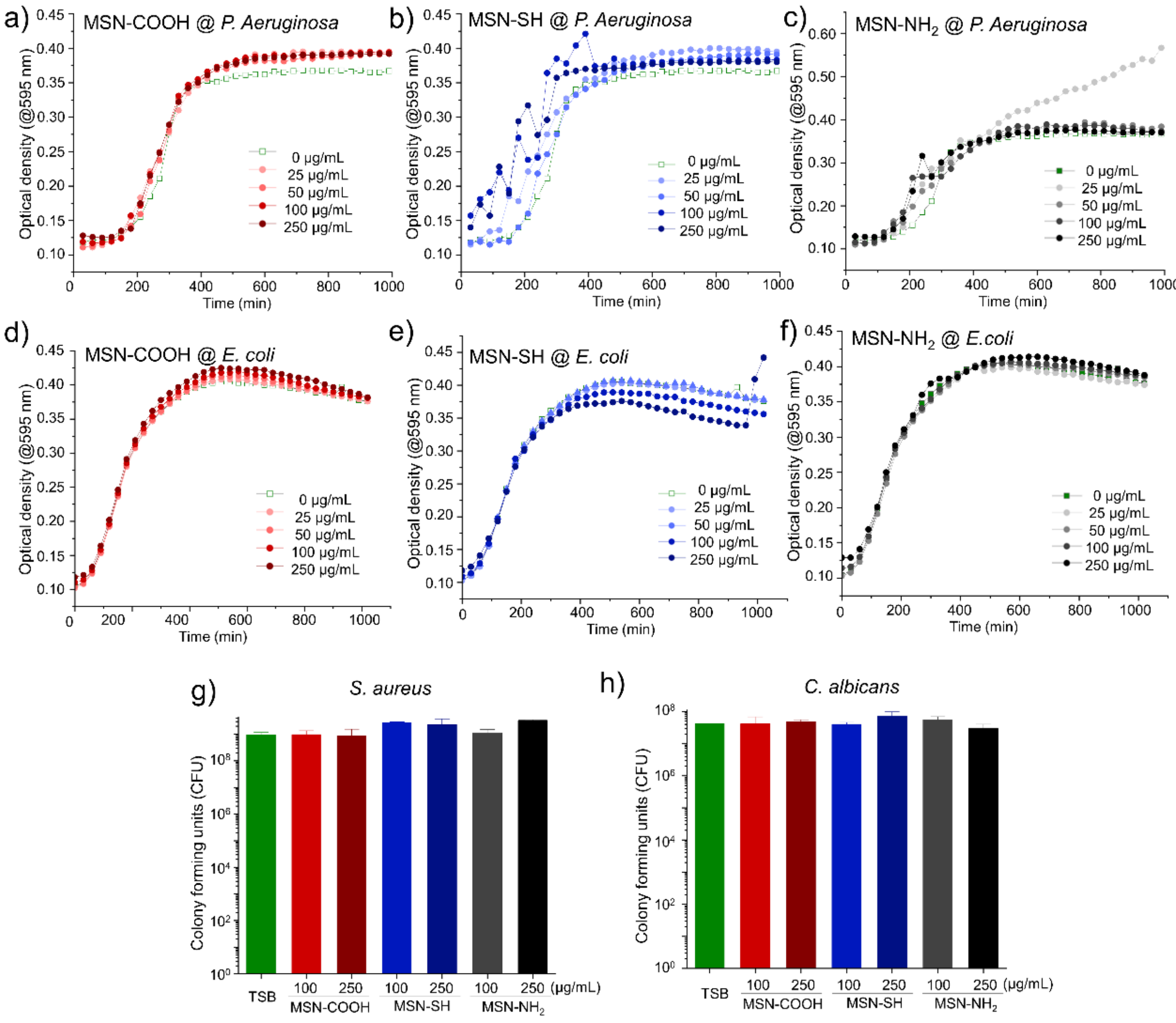

Figure S2 Antimicrobial activity of MSN without ion loading. a-c) Optical density at 595 nm of *P. aeruginosa* culture in media containing varying concentrations of MSN-COOH (a), MSN-SH (b) or MSN-NH2 (c). d-f) Optical density at 595 nm of *E. coli* culture in media containing varying concentrations of MSN-COOH (d), MSN-SH (e) or MSN-NH2 (f). g,h) Colony forming units quantified for *S. aureus* (g) and *C. albicans* (h) cultured overnight with varying concentrations of MSN-COOH, MSN-SH or MSN-NH_2 h).

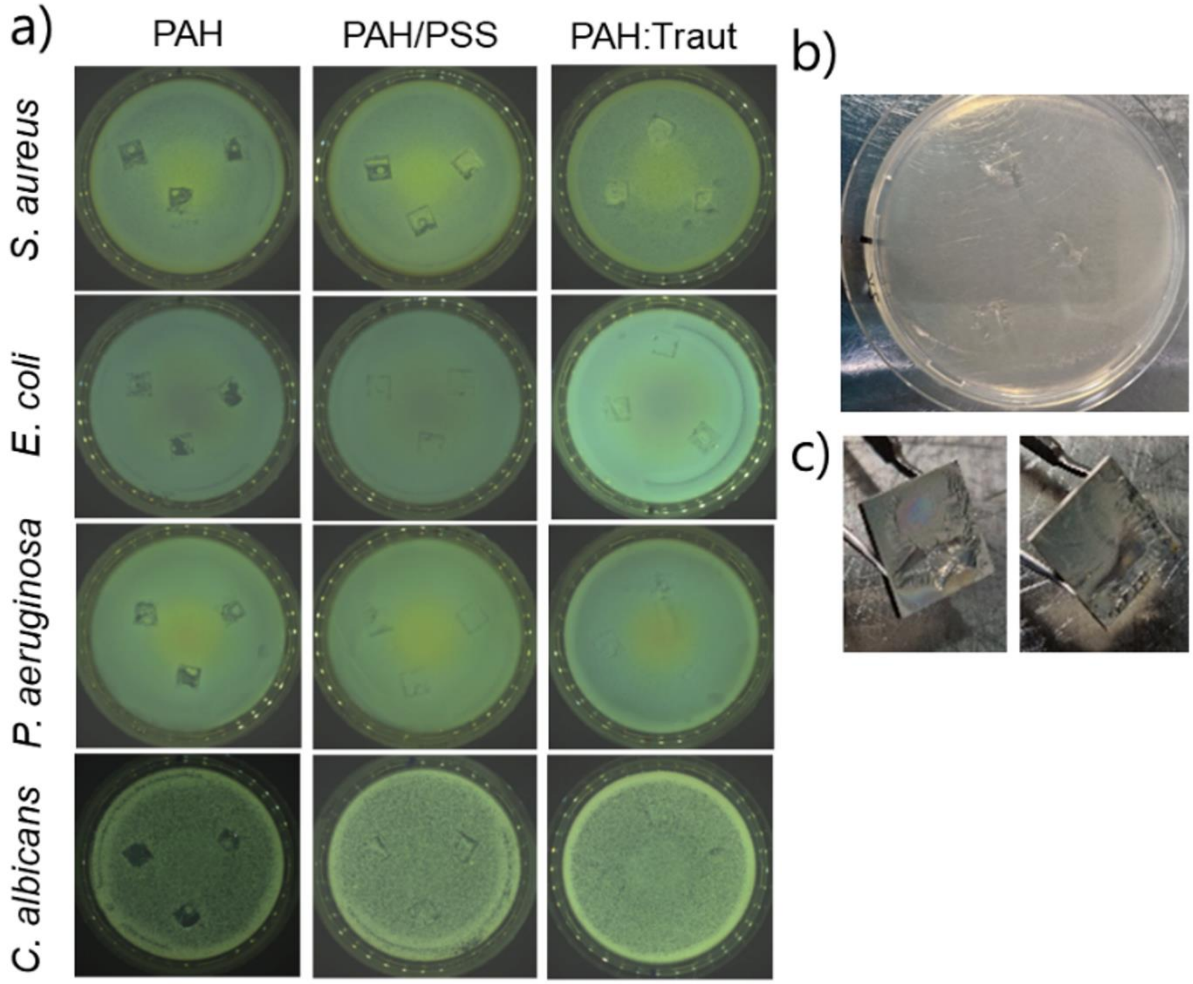

Figure S3 Touch test results for stainless steel substrates with adhesive layer coatings (a). An agar gel after the removal of PAH-coated SS (b). PAH-coated SS removed from an agar gel (c). Upon removal of the PAH-coated SS from the microbial lawn, pieces of agar gel with microbes were strongly attached to the PAH coating and removed from the culture plate. This likely explains the observed colony-free area for the PAH coating in (a). Additionally, non-antimicrobial activity of PAH/PSS and PAH:Traut coatings further support that the PAH coating alone is not antimicrobially active. b) and c) suggest that PAH itself is not antimicrobial, and the absence of microbes is rather due to the removal of parts of the agar gel than antimicrobial action. When particles cover the PAH adhesion layers, the agar gel stays (mostly) intact after removing the substrates (see Figure 5.d for MSN-COOH on PAH adhesion layers).

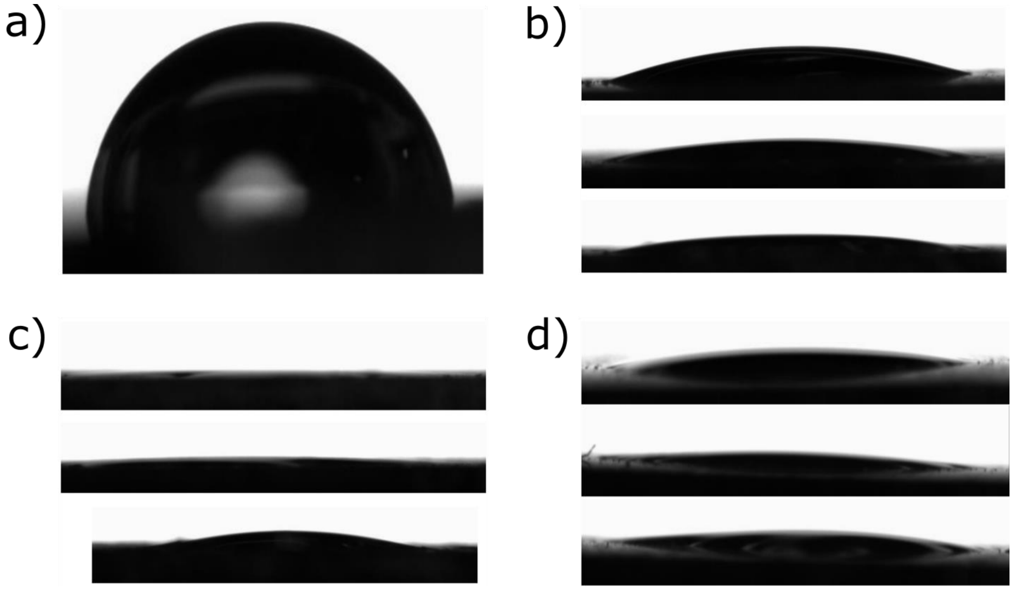

Figure S4 Water contact angles of uncoated stainless steel (a), and stainless steel coated with adhesion layers and silver-ion loaded MSN-COOH (b), MSN-SH (c), and MSN-NH2 (d).

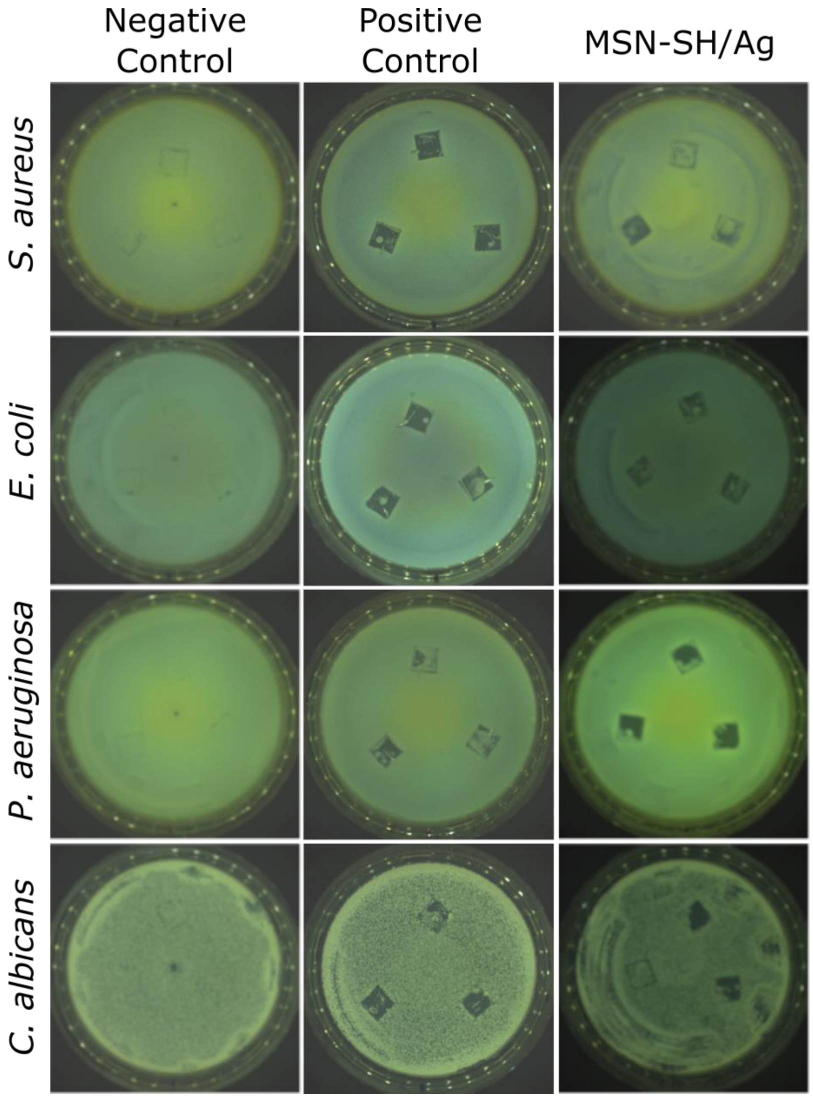

Figure S5 Touch Tests results for MSN-SH loaded with Ag on a PAH:TRAUT 2:1 adhesion layer after abrasion tests. An uncoated copper coupon that was immersed in 28% ammonia solution for over 1 h prior to use and not subjected to simulated wear is shown as the positive control here.

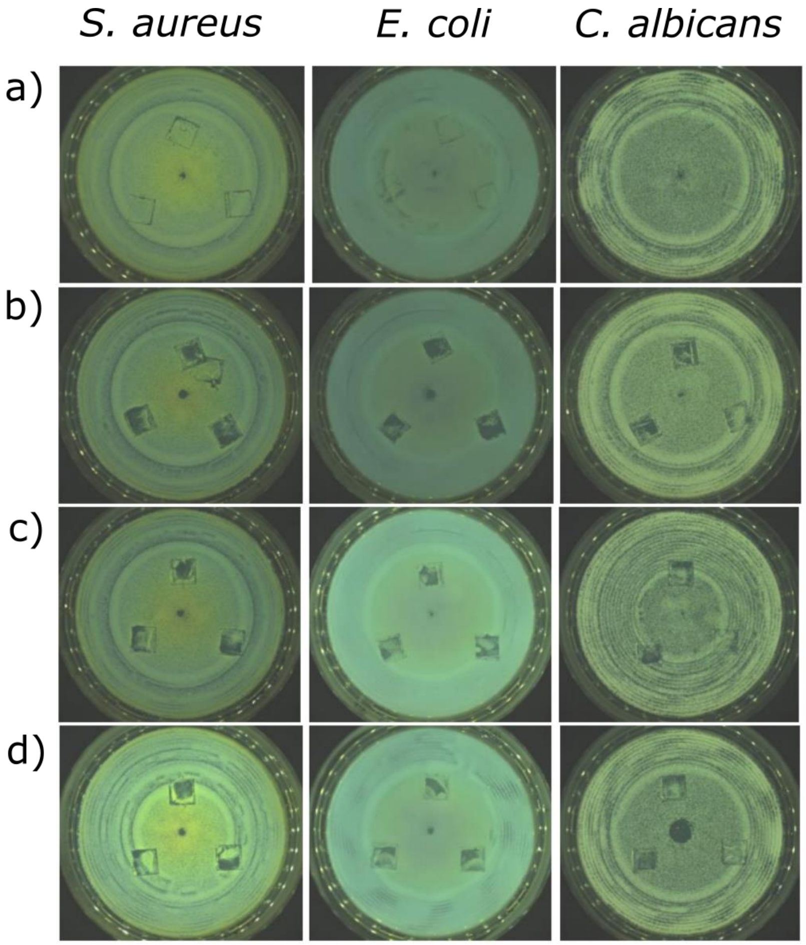

Figure S6 Repeated touch tests for MSN-SH/Ag. Uncoated stainless steel (a), and MSN-SH/Ag after 1st touch test (b), 2nd touch test (c), and 3rd touch test (d).

Table S1 Data from Nitrogen Sorption Measurements.

Property	$MSN-NH_2$	MSN-COOH	MSN-SH
S_{BET} (m^2/g)	1000	909	1161
V_{tot} (cc/g)	1.2	1.2	1.6
d_{Pore} (BJH) (nm)	3.0	3.0	3.0
d_{Pore} (NLDFT) (nm)	3.5	3.8	3.5
V_{tot} (NLDFT) (cc/g)	1.1	1.1	1.2
$V_{Mesopores}$ (NLDFT) (cc/g)	0.7	0.7	0.7
